# Supplementary material for: Poly(amidoamine) Dendrimer/Camptothecin Complex: From Synthesis to In Vitro Cancer Cell Line Studies
Source: Molecules. 2023 Mar 16;28(6):2696. doi: 10.3390/molecules28062696 (PMC10052527; doi:10.3390/molecules28062696)
Supplement: Supplementary file 1 [file molecules-28-02696-s001.zip › Table S1.pdf]

Table S1. Percentage content of carboxylic and lactone forms of CPT at pH 7.40±0.05.

	**Carboxylic form of CPT**		**Lactone form of CPT**	
**Time [h]**	**Concentration [ug/ml]**	**Content [%]**	**Concentration [ug/ml]**	**Content [%]**
1.5	30.08	83.88	5.78	16.12
3	49.20	86.33	7.65	13.67
6	71.78	88.94	8.43	11.06
8.5	71.86	89.10	8.33	10.90
21	75.58	90.64	6.83	9.36
28	64.71	90.17	6.44	9.83
48	53.74	90.09	5.43	9.91
75	47.82	90.04	4.91	9.96
98	39.10	89.77	4.35	10.23
168	32.24	88.56	4.99	11.35
