# Supplementary figures and images for: Poly(amidoamine) Dendrimer/Camptothecin Complex: From Synthesis to In Vitro Cancer Cell Line Studies
Source: Molecules. 2023 Mar 16;28(6):2696. doi: 10.3390/molecules28062696 (PMC10052527; doi:10.3390/molecules28062696)

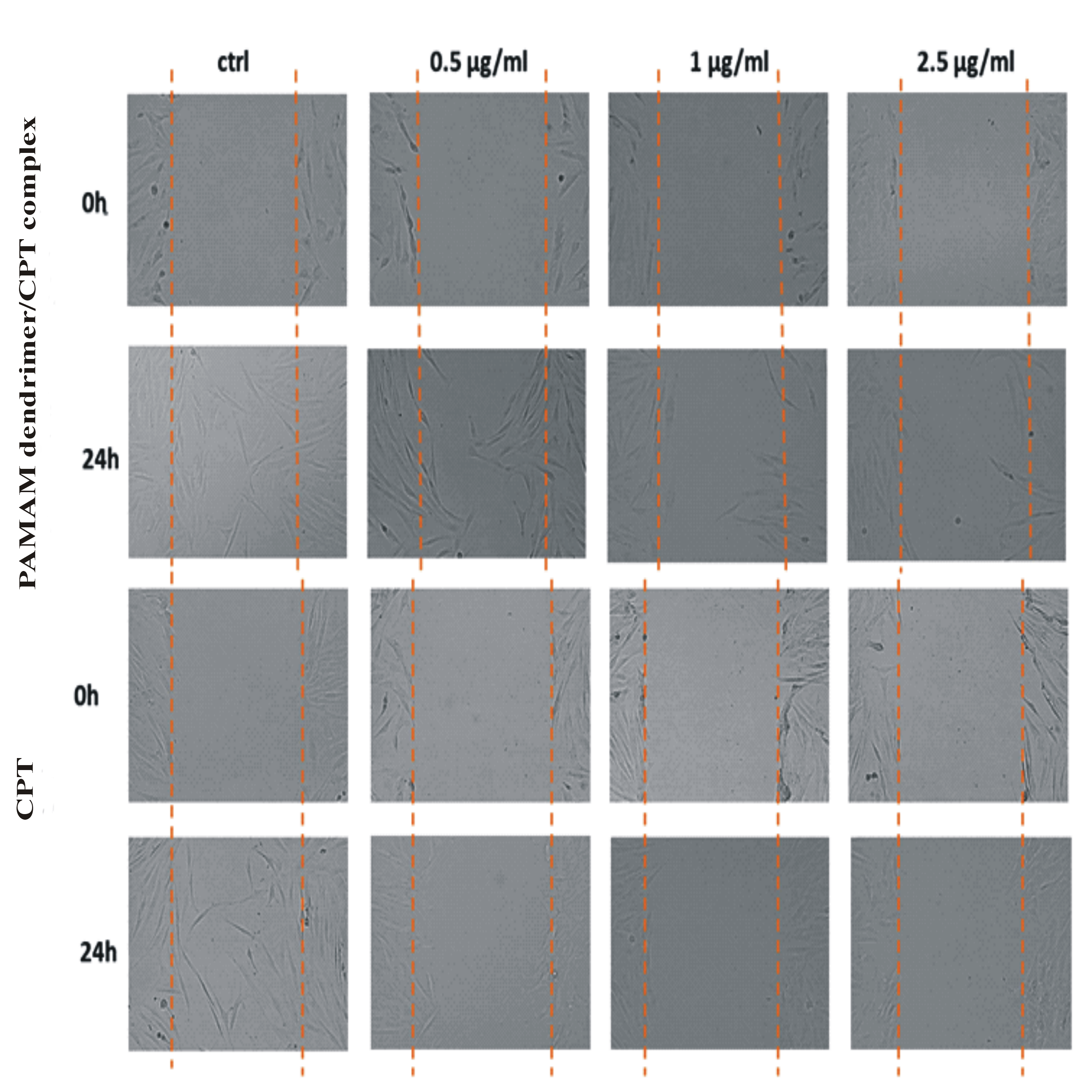

Supplement: Supplementary file 1 [file molecules-28-02696-s001.zip › Figure S10.TIF]

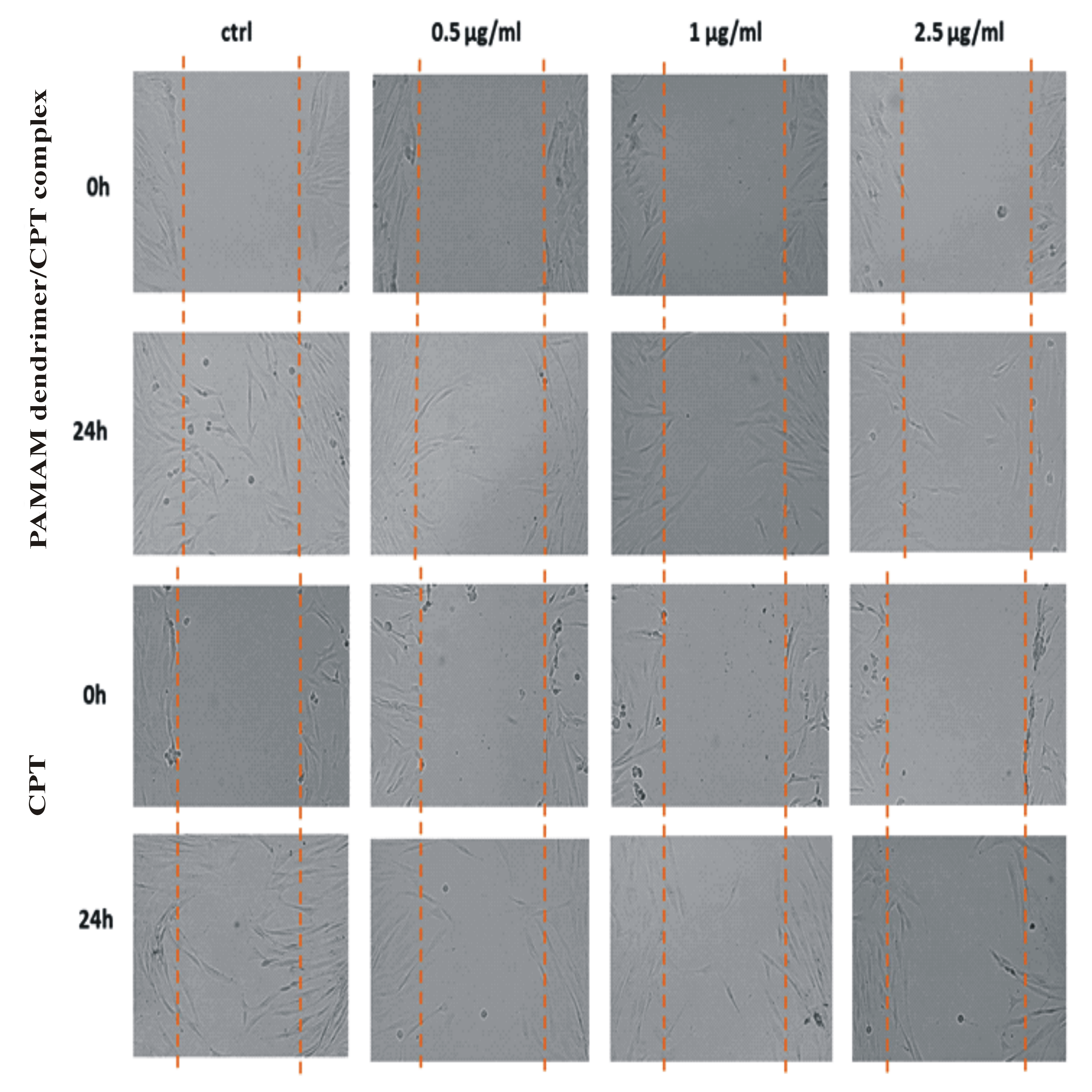

Supplement: Supplementary file 1 [file molecules-28-02696-s001.zip › Figure S11.TIF]

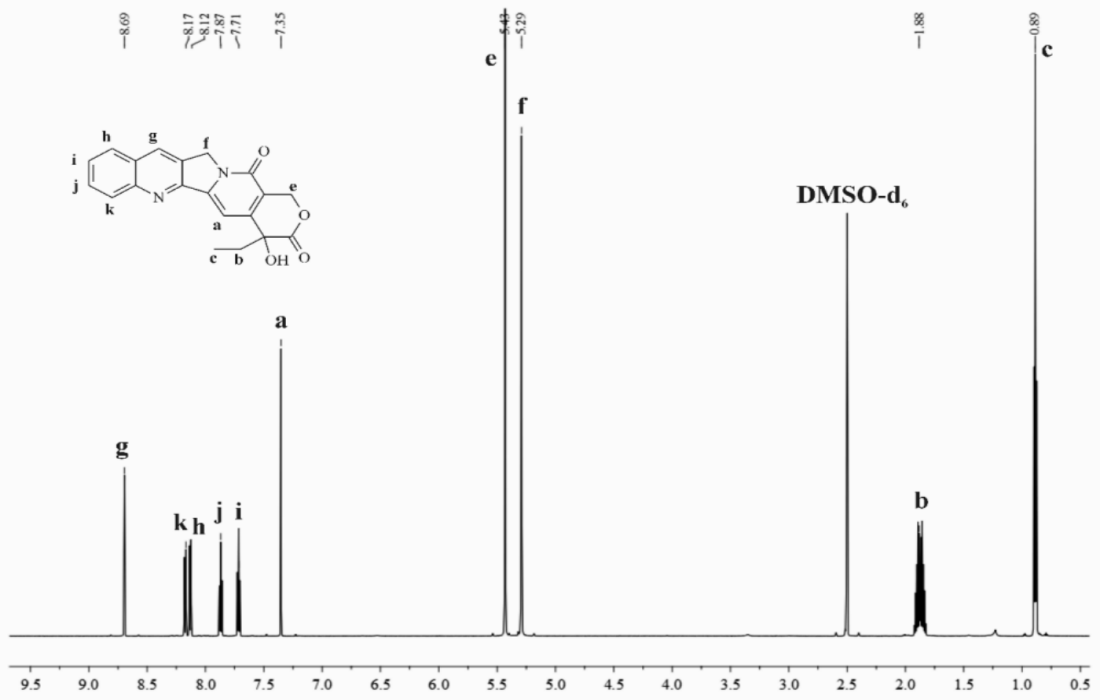

Supplement: Supplementary file 1 [file molecules-28-02696-s001.zip › Figure S2.tif]

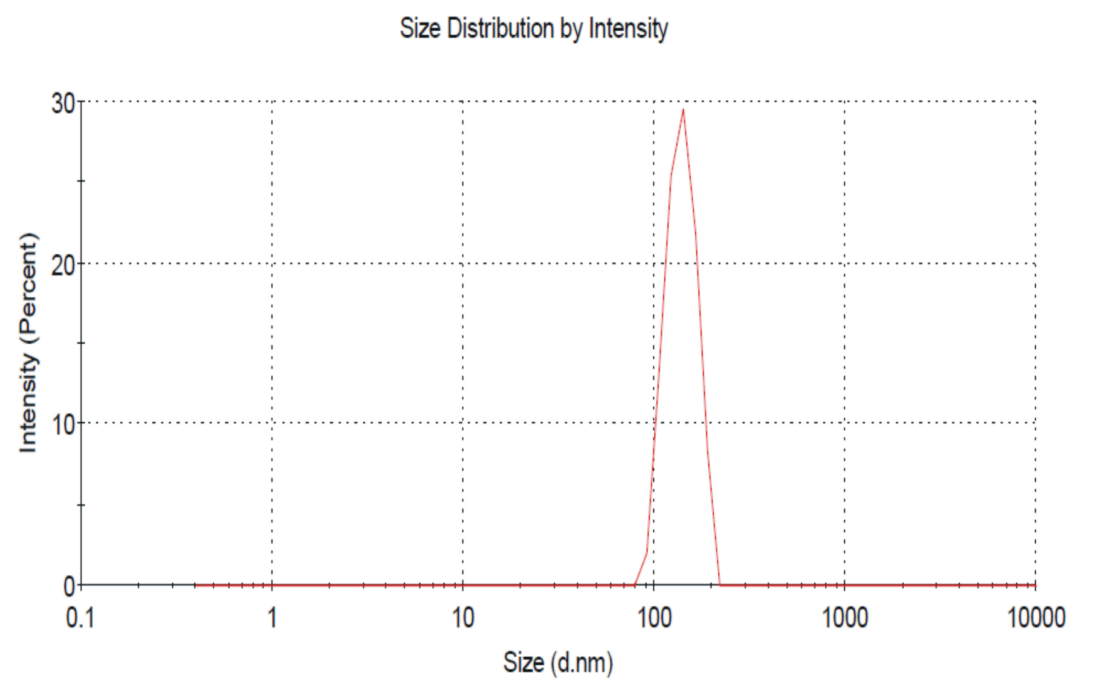

Supplement: Supplementary file 1 [file molecules-28-02696-s001.zip › Figure S4.TIF]

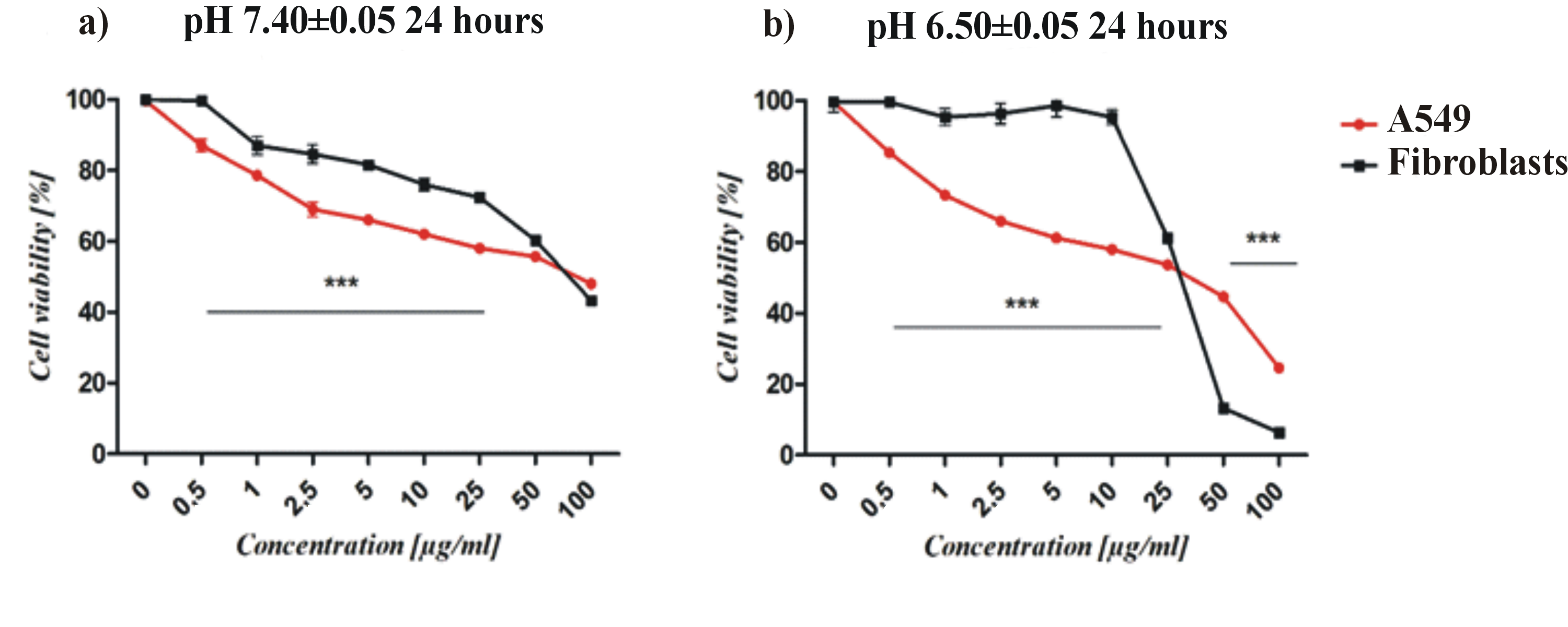

Supplement: Supplementary file 1 [file molecules-28-02696-s001.zip › Figure S5.TIF]

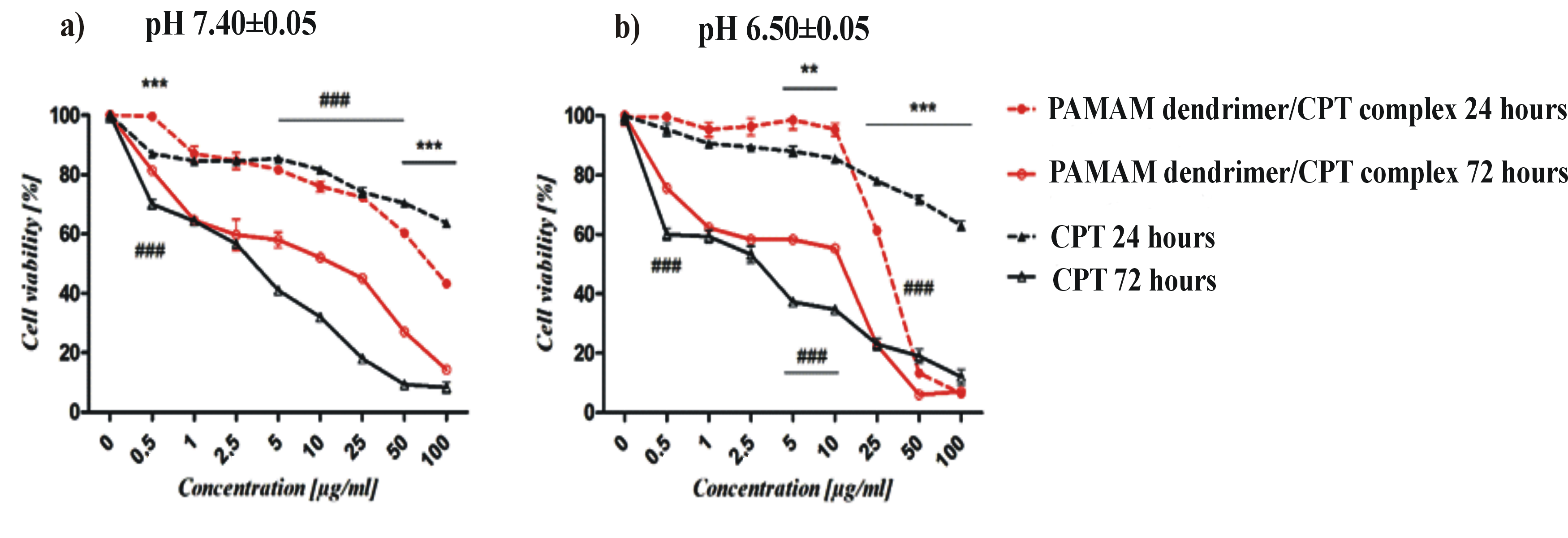

Supplement: Supplementary file 1 [file molecules-28-02696-s001.zip › Figure S6.TIF]

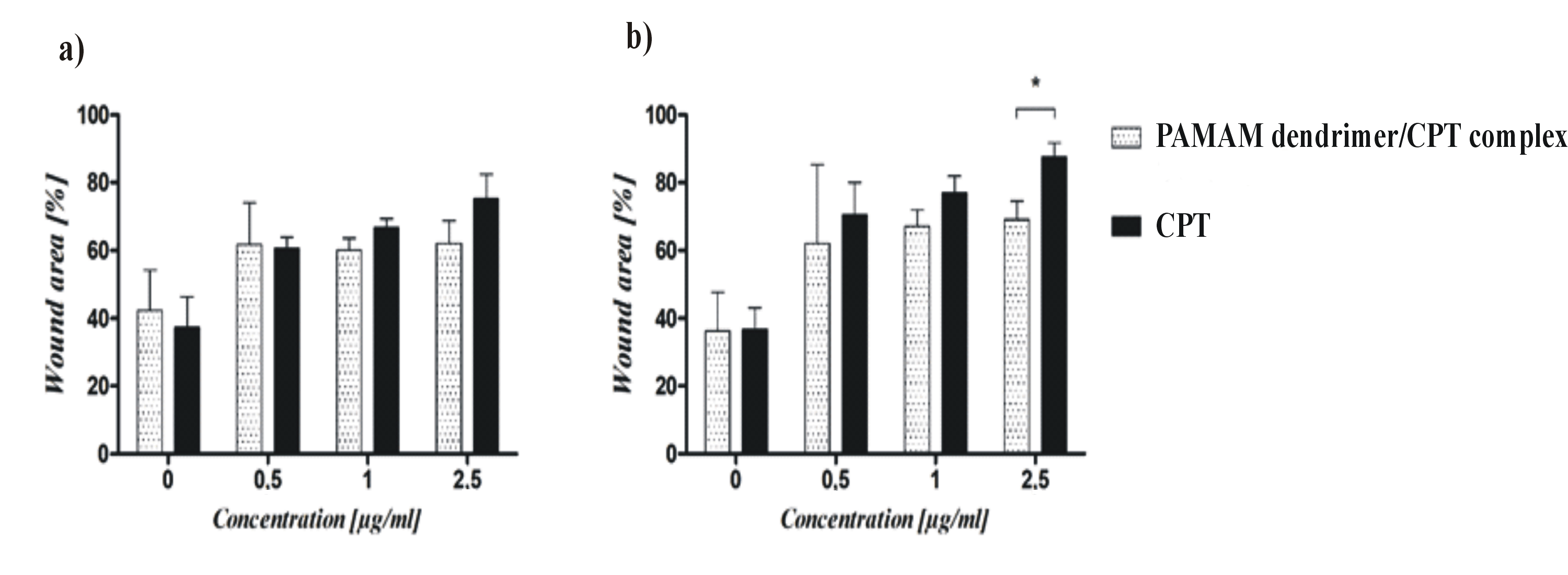

Supplement: Supplementary file 1 [file molecules-28-02696-s001.zip › Figure S7.TIF]

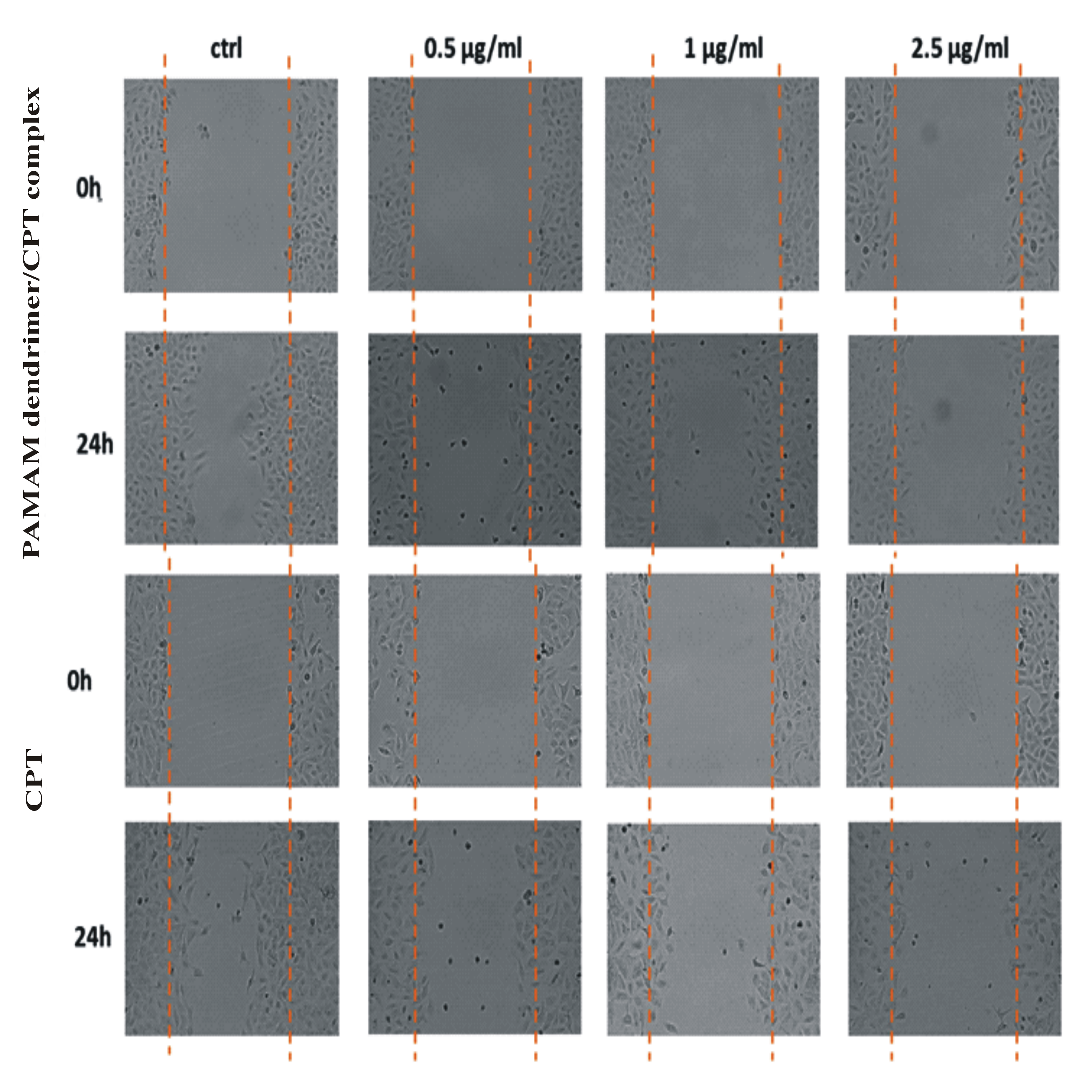

Supplement: Supplementary file 1 [file molecules-28-02696-s001.zip › Figure S8.TIF]

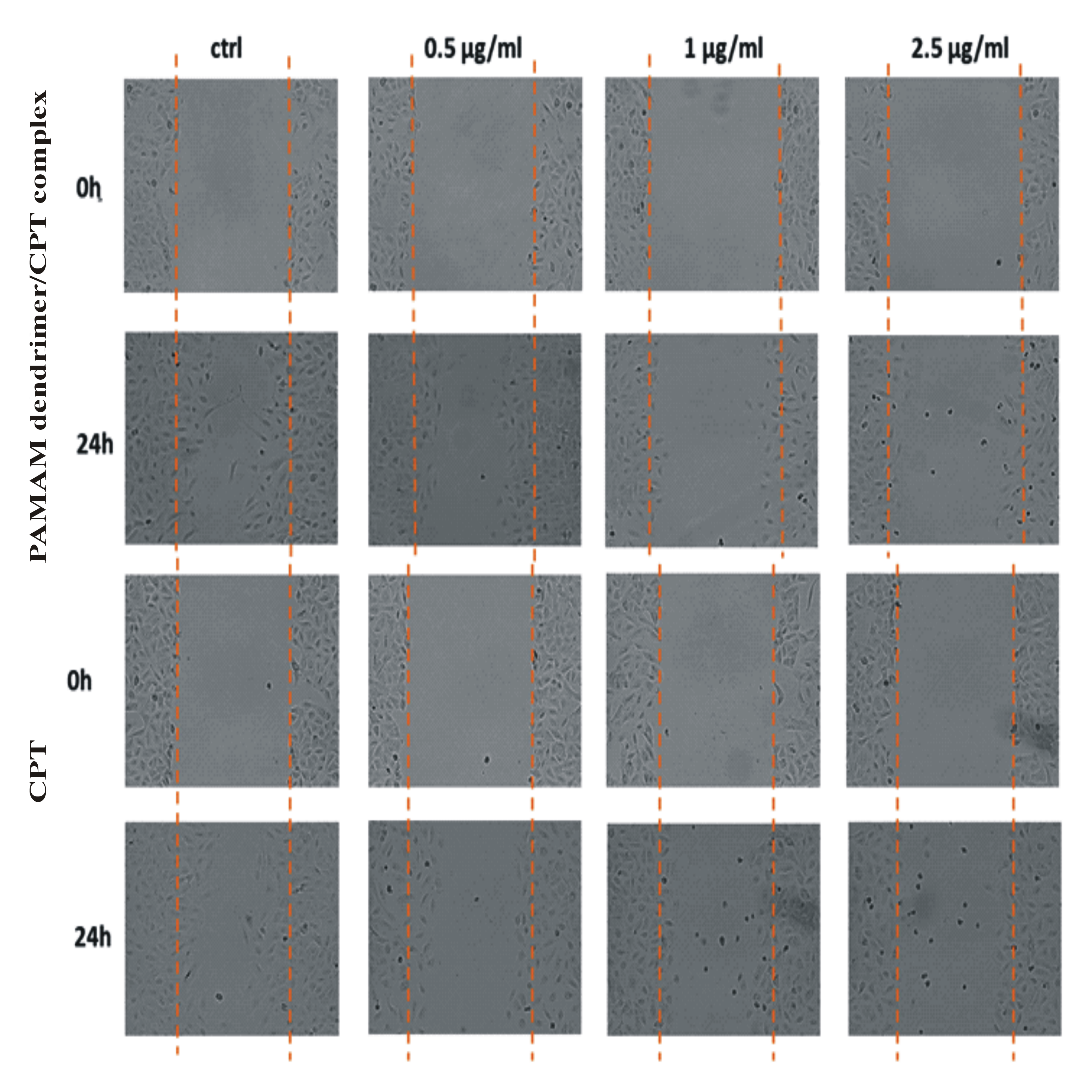

Supplement: Supplementary file 1 [file molecules-28-02696-s001.zip › Figure S9.TIF]
